# Paper Actuators Made with Cellulose and Hybrid Materials

**DOI:** 10.3390/s100301473

**Published:** 2010-02-26

**Authors:** Jaehwan Kim, Sungryul Yun, Suresha K. Mahadeva, Kiju Yun, Sang Yeol Yang, Mohammad Maniruzzaman

**Affiliations:** Center for EAPap Actuator, Department of Mechanical Engineering, Inha University, 253 Yonghyun-Dong, Nam-Gu, Incheon 402-751, Korea; E-Mails: ysy1129@inha.ac.kr (S.Y.); suresha_km@hotmail.com (S.K.M.); blackhole97@hanmail.net (S.Y.); mamun08me@yahoo.com (M.M.)

**Keywords:** electro-active polymers, smart materials, cellulose, piezoelectricity, ion migration

## Abstract

Recently, cellulose has been re-discovered as a smart material that can be used as sensor and actuator materials, which is termed electro-active paper (EAPap). This paper reports recent advances in paper actuators made with cellulose and hybrid materials such as multi-walled carbon nanotubes, conducting polymers and ionic liquids. Two distinct actuator principles in EAPap actuators are demonstrated: piezoelectric effect and ion migration effect in cellulose. Piezoelectricity of cellulose EAPap is quite comparable with other piezoelectric polymers. But, it is biodegradable, biocompatible, mechanically strong and thermally stable. To enhance ion migration effect in the cellulose, polypyrrole conducting polymer and ionic liquids were nanocoated on the cellulose film. This hybrid cellulose EAPap nanocomposite exhibits durable bending actuation in an ambient humidity and temperature condition. Fabrication, characteristics and performance of the cellulose EAPap and its hybrid EAPap materials are illustrated. Also, its possibility for remotely microwave-driven paper actuator is demonstrated.

## 1. Introduction

**Figure 1.**
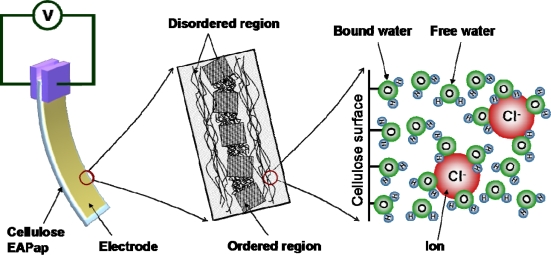
Schematic of cellulose EAPap: actuator configuration (left), illustrated structure of regenerated cellulose film (middle) and distribution of water molecules with Cl^−^ ions (right).

Cellulose is one of the most abundant natural polymers on earth [1]. Since cellulose is environmentally friendly, renewable and biocompatible material, it has been utilized for many applications including the immobilization of proteins, antibodies, coatings, artificial arteries and organs, as well as the formation of cellulose composites with synthetic polymers and biopolymers. Recently, cellulose paper has been discovered as a smart material, termed electro-active paper (EAPap), which can be used as sensors and actuators [2,3]. EAPap has many advantages in terms of lightweight, dryness, low cost, biodegradability, large deformation, low actuation voltage and low power consumption. EAPap is electrically activated due to a combination of ion migration and piezoelectric effect. Figure 1 shows the schematic of a cellulose EAPap actuator. Piezoelectric effect in cellulose has been reported a long time ago, although its effect was small [4]. Piezoelectricity in cellulose originates from the dipolar orientation and monoclinic crystal structure of cellulose. Aligned cellulose chains by using electric field, magnetic field and mechanical stretching resulted in improved piezoelectric effect in the cellulose [5–7]. Its maximum actuator performance, however, is shown at high humidity condition, and the actuator performance tends to degrade with time. As an attempt to improve the performance of the EAPap actuator, polypyrrole (PPy) and polyaniline conductive polymer coating on cellulose EAPap [8,9], mixing carbon nanotubes with cellulose [10,11], cellulose-chitosan blending [12,13] and ionic liquid blending [14,15] have been done. As a result, dry and durable EAPap actuator that can be actuated in an ambient humidity condition has been made.

In this paper, recent research activities in the cellulose EAPap material are overviewed and its possibility for remotely-driven paper actuator is demonstrated. Cellulose EAPap is customized, namely piezo-paper, by maximizing its piezoelectricity. Characteristics and performance of piezo-paper are explained in this paper. On the other hand, to further enhance the ion migration effect in EAPap, carbon nanotubes blending with cellulose as well as nanocoating of PPy and ionic liquids are illustrated. Remotely-driven paper actuator is made with cellulose EAPap on which rectifying antenna (rectenna) array is fabricated [16]. Rectenna can convert microwave power into DC power that can be used for activating EAPap actuators. Microwave power transmission using rectenna has broad technological impact in many applications, for example, remote sensors, remotely-driven flying objects, power transmission for biomedical devices and remote power supply for information technology devices [17]. The fabrication of some of these devices, along with the characterization and their demonstrations are illustrated.

## 2. Results and Discussion

### 2.1. Piezo-paper Bending Actuator

**Figure 2.**
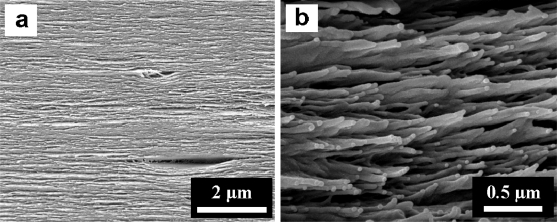
Cross-sectional morphology of regenerated cellulose film taken by scanning electron microscopy (SEM): a) before stretching and b) after stretching (S_R_ = 1.6).

Regenerated cellulose film was fabricated by curing cellulose solution prepared by homogeneous reaction in DMAc/LiCl solvent system [18]. Piezo-paper has been made by manually stretching the regenerated cellulose film in a wet state [19]. Generally, the regenerated cellulose film shows layer-by-layer structure composed of hydrogen bond networks among cellulose chains [20]. It is known from our previous research that mechanical stretching of wet cellulose film produces an aligned nanofiber structure (diameter: 30–150 nm) of cellulose chains. This alignment predominantly contributed to enhancing the mechanical and piezoelectric characteristics of regenerated cellulose film [19]. In this study, we fabricated piezo-paper by using an automated process. Regarding the fibrous structure of the piezo-paper, its structural morphology was similar to the manually made one (Figure 2). However, the automation process is more efficient than the manual process to improve the mechanical and piezoelectric characteristics. In terms of piezoelectric property, the piezo-paper made by the automated process showed predominant piezoelectric charge constant [d_31_] compared with that of the manually made one. Note that the d_31_ value of 45° direction was 40% higher than that of 0° orientation sample (Table 1). This indicates that the automated casting and stretching processes can allow stable and enhanced mechanical and piezoelectric properties of piezo-paper [21].

**Figure 3.**
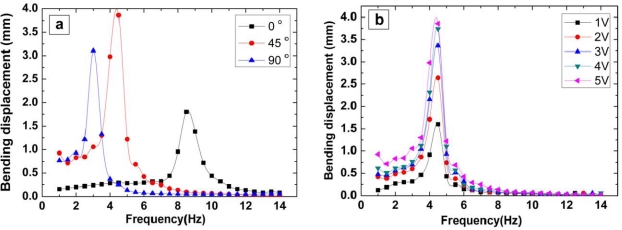
Actuator performance of piezo-paper bending actuator depending on a) orientation and b) applied voltage (Thickness: 22 μm, Young’s modulus: 8.9 GPa (0°), 3.5 GPa (45°), 2.0 GPa (90°)).

To evaluate the actuator performance of the piezo-paper, bending displacement and resonance frequency of the piezo-paper bending actuator were measured depending on the orientation angle and applied voltage at 90% relative humidity (RH) (Figure 3). The stretching ratio was 1.6. The 45° orientation sample showed the best actuation performance (Figure 3a), and the bending displacement increased as the applied voltage increased (Figure 3b).

The actuation principle of piezo-paper can be explained in terms of ion migration and shear piezoelectricity of cellulose. Since we dissolved the cellulose in LiCl/DMAc solvent system and regenerated cellulose film was prepared by curing in DI water/IPA solvent mixture to effectively eliminate Li^+^ (DMAc)_x_ macrocations, the piezo-paper actuator has been known to have small amount of remnant Cl^−^ ions [18]. Thus, when the actuator was excited with electric field at 90% RH, the mobile Cl^−^ ions in such a humid condition contributed to generate a large bending displacement. Furthermore, the actuator has shear piezoelectricity, which shows its maximum at 45° orientation angle [21]. This shear piezoelectricity of piezo-paper can be effectively shown at low humidity condition.

### 2.2. MWCNT-Cellulose Hybrid Nanocomposite

**Figure 4.**
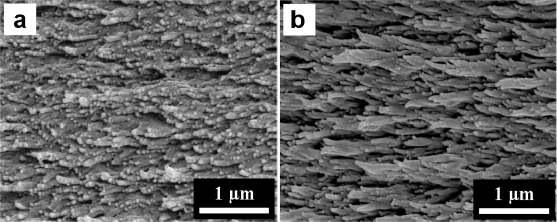
SEM cross-sectional morphology of: a) cellulose film, b) M/C composite and c) aligned M/C composite.

Multi-walled carbon nanotubes (MWCNTs) covalently grafted cellulose (M/C) hybrid nanocomposite was fabricated, and mechanically stretched to align MWCNTs with cellulose. Characteristics of the M/C hybrid nanocomposite were evaluated by observing its morphology, Young’s modulus and electrical resistance. Figure 4 shows the cross-sectional morphologies of the M/C hybrid nanocomposite taken by SEM. By covalently grafting MWCNTs on cellulose chains, MWCNTs were homogeneously distributed in cellulose matrix, but the cellulose layered structure disappeared. This indicates that the covalent grafting of MWCNTs on cellulose chains can disturb the formation of hydrogen bond networks between the cellulose chains, whereas it can be a good method to prevent aggregation of MWCNTs in cellulose matrix. Table 2 shows the Young’s modulus and electrical resistance of the hybrid nanocomposites before and after mechanical stretching compared with that of cellulose. The results reveal that the mechanical stretching effectively improved the mechanical and electrical properties of M/C hybrid nanocomposite.

**Figure 5.**
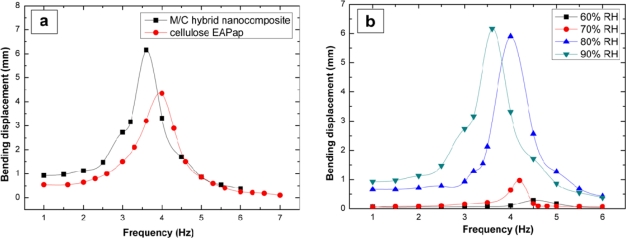
Actuator performance of M/C hybrid nanocomposite: a) compared with cellulose EAPap and b) depending on humidity level (Thickness (μm)/Young’s modulus (GPa): 16/6.0 (60% RH), 19/3.2 (70% RH), 21/2.9 (80% RH), 22/2.4 (90% RH)).

The M/C hybrid nanocomposite was tested for a bending actuator by depositing electrodes on both sides of the composite. Its actuator performance was evaluated by measuring bending displacement and resonance frequency with different humidity levels. M/C hybrid nanocomposite exhibited larger bending displacement at lower resonance frequency than that of cellulose EAPap actuator (Figure 5a). As the humidity level increased from 60 to 90% RH, the bending displacement increased, while the resonance frequency moved to a lower band (Figure 5b). This humidity-sensitive behavior might be associated with the presence of many hydrophilic functional groups on cellulose chains as well as covalently grafted MWCNTs. The actuation principle of M/C hybrid nanocomposite is similar to the ion migration of piezo-paper (cellulose EAPap) actuator. However, despite the same experimental conditions, the bending displacement of M/C hybrid nanocomposite is much larger than that of cellulose EAPap. This might be due to further expansion of positive electrode side by interaction of Cl^−^ ions with covalently grafted MWCNTs, and the increased cellulose chain mobility caused by weakened intra-intermolecular hydrogen bonds among cellulose chains [24].

### 2.3. Ionic liquid-PPy-Cellulose Hybrid Nanocomposite

**Figure 6.**
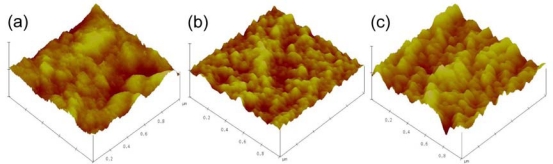
Tapping mode AFM images of: a) pristine cellulose, b) PPy coated cellulose and c) ionic liquid-PPy-cellulose hybrid nanocomposite.

To further develop robust cellulose EAPap bending actuator, ionic liquid and PPy were nanocoated on cellulose film. Formation of ionic liquid-PPy-cellulose hybrid nanocomposite was assessed by atomic force microscopy (AFM) and X-ray photoelectron spectroscopy (XPS). Figure 6 shows the tapping mode AFM images of pristine cellulose, PPy coated cellulose and ionic liquid-PPy-cellulose hybrid nanocomposite. In order to enumerate the formation of nanoscaled PPy and ionic liquid layer over cellulose surface, we measured the roughness of the samples. RMS (R_q_) value for pristine cellulose was found to be 4.2 ± 0.3 nm and this value was changed to 4.9 ± 0.7 nm and 6.2 ± 0.4 nm, for PPy coated cellulose and ionic liquid-PPy-cellulose hybrid nanocomposite, respectively.

**Figure 7.**
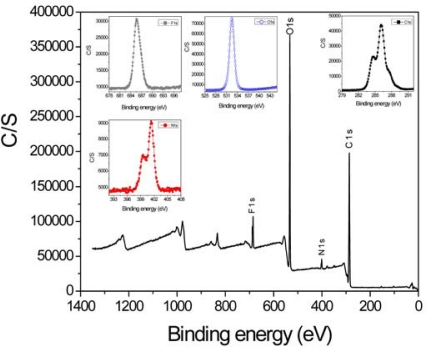
XPS survey spectra of ionic liquid-PPy-cellulose hybrid nanocomposite: insets show the detailed C1s, N1s, O1s and F1s spectra.

Figure 7 illustrates the XPS survey spectra of ionic liquid-PPy-cellulose hybrid nanocomposite. Insets of Figure 7 show the detailed C1s, N1s, O1s and F1s spectra of the nanocomposite. Pristine cellulose shows only carbon and oxygen peaks at 286.08 and 532.48 eV [22], while new peaks at 401.38 eV (N1s) and 685.58 eV (F1s) suggest the presence of nanoscaled PPy and ionic liquid on the cellulose film surface. Above changes in the RMS (R_q_) values and XPS spectra indicate the successful formation of ionic liquid-PPy nanolayer on the cellulose film.

**Figure 8.**
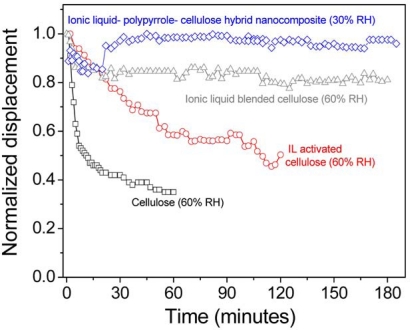
Compared durability of EAPap actuators.

Bending displacement of the ionic liquid-PPy-cellulose hybrid nanocomposite actuator was measured. Displacements of the actuators based on pristine cellulose, ionic liquid activated cellulose and ionic liquid blended cellulose are also included for comparison. It was found that the pristine cellulose exhibited 0.6 mm of displacement output at 60% RH and this value was enhanced to 3.2 mm and 5.3 mm, when the cellulose was activated and blended with ionic liquid, respectively under the same test condition. On the other hand, the introduction of nanoscaled PPy and ionic liquid layer on the cellulose film drastically improved its actuator performance: 10 mm of bending displacement at 30% RH. Durability of the actuator was tested by actuating it continuously for a long time. Figure 8 shows the result. Other actuators were included for comparison. Actuator performance degradation of the pristine cellulose actuator was found to be 60%, whereas the ionic liquid activated and ionic liquid blended cellulose actuators were 50% and 18%, respectively. Interestingly, the ionic liquid-PPy-cellulose hybrid nanocomposite actuators showed excellent durability without any actuator performance degradation for 180 minutes. The superior actuator performance and durability of ionic liquid-PPy-cellulose hybrid nanocomposite actuators might be due to the presence of nanoscaled PPy intermediate layer between cellulose and ionic liquid nanolayer and the formation of uniform and stable ionic liquid nanolayer over the cellulose-PPy surface through ionic interaction, thereby eradicates ion depletion and electrode damage.

### 2.4. Remotely Microwave-Driven EAPap Actuator

**Figure 9.**
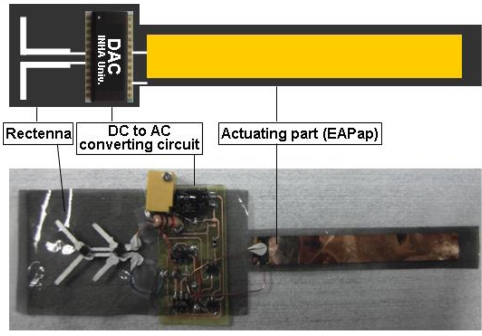
Configuration of Microwave driven EAPap actuator.

Microwave driven EAPap actuator is attractive for many actuator applications since it does not need to carry a battery. We fabricated a microwave-driven EAPap actuator by fabricating rectenna and DAC (DC to AC converting circuit) with ionic liquid-PPy-cellulose EAPap actuator. Figure 9 shows its schematic diagram and prototype. Rectenna is a rectifying antenna that can convert microwave power into dc power. Rectenna consists of dipole micro-strip antenna and Schottky diode.

**Figure 10.**
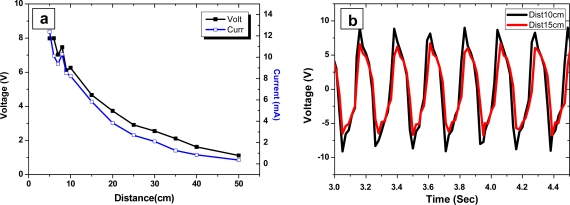
Voltage and current output of rectenna: a) with distance before DAC, b) voltage time signal after DAC.

DC power was remotely generated on the actuator through the rectenna by sending 1.2 W microwave power. Generated voltage and current are shown in Figure 10a. Maximum 8 V and 12 mA were obtained at near field. Figure 10b shows the voltage time signal after the DAC. This voltage is enough to active the EAPap actuator.

## 3. Experimental Section

### 3.1. Piezo-Paper Fabrication

The cotton pulp (Buckeye) with the degree of polymerization (DP), 4500 and LiCl (Junsei Chemical) was dried in an oven at 100 °C to evaporate absorbed water molecules. The cutton pulp was mixed with LiCl/anhydrous DMAc (Aldrich) in proportion to cotton cellulose pulp/LiCl/DMAc (2/8/90). The cellulose was dissolved in the solvent system by heating at 155 °C with mechanical stirring followed by the solvent exchange technique [23]. The cellulose solution was centrifuged at 11000 rpm to eliminate undissolved cellulose fibers.

**Figure 11.**
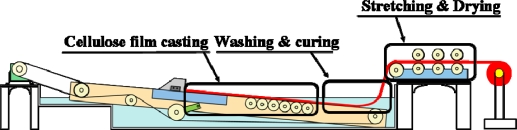
Configuration of mass production system of piezo-paper.

The centrifuged cellulose solution was cast on a belt using a doctor blade and cured with DI/IPA mixture, followed by the stretching/drying process. Figure 11 shows the automated process for the Piezo-paper fabrication. For curing, mist of curing solvent that consists of deionized (DI) water/Isopropyl alcohol (IPA) was sprayed. The resultant gel-like cellulose film was slowly immersed into the solvent mixture (DI/IPA) bath to effectively eliminate Li^+^ ions as well as DMAc [18]. After rinsing the film with DI water, it was mechanically stretched via zone-stretching process to align cellulose chains with in-plane direction. Continuous casting, curing and stretching/drying were made.

To evaluate characteristics of the piezo-paper, Young’s modulus, piezoelectric charge constant, SEM morphology were investigated. The actuator performance was investigated by measuring its bending displacement and resonance frequency. A previously developed computerized actuator performance measurement system [10] was used.

### 3.2. MWCNT-Cellulose Hybrid Nanocomposite

The cellulose solution process is the same as in the previous section. MWNTs (Aldrich) were functionalized with carboxyl groups and hydroxyl groups by nitric acid treatment [24] and filtered with deionized (DI) water until the pH reached 7. The functionalized MWNTs (6 mg) were dispersed in DMAc (10 g) by sonication in an ultrasonic bath (Fisher, FS30H) for 10 min. CDI (120 mg) was dissolved in DMAc (10 g). The solutions were mixed and sonicated in an ultrasonic bath at 60 °C for 12 hours. The reaction resulted in converting carboxyl groups on MWNTs to imidazolide. By reacting imidazolide-MWNTs solution with cellulose solution at 60 °C for 18 hours, MWNTs were covalently bonded to cellulose molecules. The solutions were spin-coated on the silicon wafer and cured in order by IPA, DI water/IPA (ratio of 4:6) and DI water. This slow curing process was performed to prevent instant aggregation of MWNTs and to eliminate remnant Li^+^ (DMAc)_x_ macrocations and solvents [18]. To align covalently bonded MWNTs with cellulose chains, the wet M/C composites were mechanically stretched. During the stretching process, the films were dried with a near infra-red ray heater.

### 3.3. Ionic liquid-PPy-Cellulose hybrid nanocomposite

Cellulose solution was made according to the previous method. Thin regenerated cellulose films were obtained by spin-coating the cellulose solution on a wafer, followed by washing with DI water/IPA mixture. Nanoscaled PPy was introduced onto the wet regenerated cellulose films by employing polymerization-induced adsorption process [25]. In a typical procedure, CuCl2·2H2O solution was added drop-wise into a pyrrole solution and stirred. The reaction mixture turned black within a few minutes, gradual formation of a black PPy precipitate starts and the polymerization process was allowed for 30 min. Finally reaction mixture was filtered to remove the bulk PPy, and clear filtrate (a very dilute polymerization solution) was used for the PPy coating on the regenerated cellulose films. Wet regenerated cellulose film was immersed in 50 ml of filtered solution for 16 hours. In-situ polymerized PPy in the presence of wet cellulose film resulted in deposition of PPy on the cellulose surface. After the polymerization process, the films were washed with 2-propanol and pure water. Finally, ionic liquid-PPy-cellulose hybrid nanocomposite was prepared by activating wet PPy-cellulose films with an ionic liquid (1-butyl-3-methylimidazolium tetra fluroborate, BMIBF4) for 48 hours. Ionic liquid-PPy-cellulose hybrid nanocomposite was then dried under ambient conditions.

### 3.4. Remotely Microwave-Driven EAPap Actuator

Rectenna is composed dipole of micro-strip antenna and Schottky diode. Dipole micro-strip antenna was fabricated on a cellulose film using silk printing process. To fabricate the dipole micro-strip antenna, silver paste was spread on a silk pattern and squeezed through it to the cellulose film. The patterned silver paste was dried. Schottky diodes were connected to the fabricated antenna pattern using the silver paste. Schottky diode characteristics are important in deciding the performance of rectenna. In this paper, commercial Schottky diode (MA2054-1141T, M/A-COM) was used.

**Figure 12.**
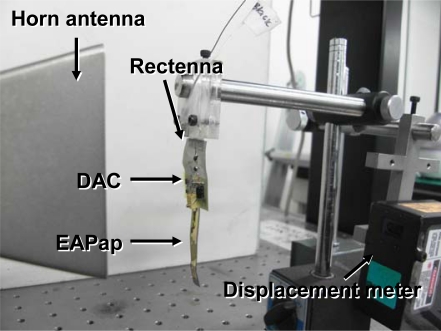
Microwave driven EAPap actuating test setup.

To evaluate the performance of microwave-driven EAPap actuator, a microwave test setup was established (Figure 12). The sample was located ∼10–15 cm away from a microwave horn antenna that transmits microwave power, and a displacement sensor was located on the opposite side. Rectenna received microwaves and converted them into DC power, and DAC converted the DC power to an alternating power so as to activate the EAPap actuator. The EAPap actuator moved according to the alternating power.

## 4. Conclusions

Piezo-paper was fabricated by using an automated process and its characteristics and actuator performance were investigated. Its mechanical and piezoelectric properties were stable and improved. Its actuation principle was found to be associated with piezoelectric effect and ion migration effect in cellulose. To further enhance the ion migration effect in the piezo-paper (cellulose EAPap), carbon nanotubes were covalently grafted with cellulose, and PPy conducting polymer as well as ionic liquids were nanocoated on the cellulose. The blending of carbon nanotubes drastically improved its Young’s modulus as well as electrical conductivity. The ionic liquid-PPy-cellulose nanocomposite exhibited durable bending actuation in the ambient humidity and temperature condition. To demonstrate the possibility of remotely microwave-driven paper actuator, rectenna was fabricated on a cellulose film, and the actuator was tested by applying remote microwave power. Since cellulose is biodegradable and biocompatible, cellulose based paper actuators can be used for biomimetic robots, ultralight weight actuators and biomedical applications.

## Figures and Tables

**Table 1.** Characteristics of piezo-paper depending on orientation and manufacturing process.

Material property	Orientation of piezo-paper					
	0°		45°		90°	

	Manu	Auto	Manu	Auto	Manu	Auto
Young’s modulus (GPa)	17.5	18.3	5.4	6.4	4.49	4.02
Piezoelectric charge constant (pC/N)	5.9	22	10.7	30.6	1.88	10.7

* Manu: Manual process, Auto: Automated process

**Table 2.** Mechanical and electrical properties of M/C hybrid nanocomposite compared with that of regenerated cellulose film.

Material	Young’s modulus [GPa]	Electrical resistance [kΩ]
Regenerated cellulose film	5.3	-
M/C hybrid nanocomposite	11.4	84.9 × 10^3^
Aligned M/C hybrid nanocomposite	23.4	107.7
